# An analysis of screen-detected invasive cancers by grade in the English breast cancer screening programme: are we failing to detect sufficient small grade 3 cancers?

**DOI:** 10.1007/s00330-020-07276-9

**Published:** 2020-09-30

**Authors:** R. G. Blanks, M. G. Wallis, R. J. Alison, R. M. Given-Wilson

**Affiliations:** 1 Cancer Epidemiology Unit, Nuffield Department of Population Health, Oxford University, Richard Doll Building, Roosevelt Drive, Oxford, OX3 7LF UK; 2 MBCHB Cambridge Breast Unit, and NIHR Cambridge Biomedical Research Centre, Cambridge University Hospitals NHS Trust, Cambridge, UK; 3 Department of Radiology, St Georges University Hospital Foundation Trust, London, UK

**Keywords:** Breast cancer, Screening, Mammography, Sensitivity, cancer grade

## Abstract

**Objective:**

Randomised controlled trials have shown a reduction in breast cancer mortality from mammography screening and it is the detection of high-grade invasive cancers that is responsible for much of this effect. We determined the detection rates of invasive cancers by grade, size and type of screen and estimated relative sensitivities with emphasis on grade 3 detection.

**Methods:**

This observational study analysed data from over 11 million screening episodes (67,681 invasive cancers) from the English NHS breast screening programme over seven screening years 2009/2010 to 2015/2016 for women aged 45–70.

**Results:**

At prevalent (first) screens (which are unaffected by screening interval), the detection rate of small (< 15 mm) invasive cancers was 0.95 per 1000 for grade 1, but for grade 3 only 0.30 per 1000. The ratio of small (< 15 mm) to large (≥ 15 mm) cancers was 1.8:1 for grade 1 but reversed to 0.5:1 for grade 3. We estimated that the relative sensitivity for grade 3 invasive cancers was 52% of that for grade 1 and the relative sensitivity for small (< 15 mm) grade 3 only 26% of that for small (< 15 mm) grade 1 invasive cancers.

**Conclusions:**

Sensitivity for small grade 3 invasive cancers is poor compared with that for grade 1 and 2 invasive cancers and larger grade 3 malignancies. This observation is likely a limitation of the current technology related to the absence of identifiable mammographic features for small high-grade cancers. Future work should focus on technologies and strategies to improve detection of these clinically most significant cancers.

**Key Points:**

*• The detection of small high-grade invasive cancers is vital to reduce breast cancer mortality.*

*• We estimate the sensitivity for small grade 3 invasive cancers may be only 26% of that of small grade 1 invasive cancers. This is likely to be associated with the non-specific mammographic features for these cancers.*

*• New technologies and appropriate strategies using current technology are required to maximise the detection of small grade 3 invasive cancers.*

**Electronic supplementary material:**

The online version of this article (10.1007/s00330-020-07276-9) contains supplementary material, which is available to authorized users.

## Introduction

Breast cancer is the world’s second largest cause of female cancer death after lung cancer despite the introduction of breast cancer screening and improved treatment. Whilst the benefits of screening in terms of mortality reduction have been accepted, there is still disagreement over the magnitude of the effect [1–3]. This also applies to overdiagnosis and the competing effects of screening and early symptomatic detection in the light of modern treatment [4–6].

Many of the UK screening programme performance measures are based on the Swedish Two County (STC) randomised controlled trial which demonstrated the importance of detecting small-grade 3 invasive cancers [7]. Porter [8] found that most interval cancers (those clinically presenting between screens) are grade 3 or grade 2 and that only a small proportion of interval cancers are grade 1. Perron [9] has similarly shown that the sensitivity of screening in Quebec is lowest for the most aggressive phenotypes and that this effect is mediated by grade. This reinforces the STC message that at least 30% of the screen-detected grade 3 invasive cancers should be < 15 mm in diameter [10] when they are less likely to have nodal involvement.

New digital technology has increased the detection rate of high-grade DCIS, grade 1 invasive cancers and grade 2 invasive cancers, but has not impacted the grade 3 detection [11]. To date in England, we have no data on change in interval cancers related to the introduction of digital mammography. Tomosynthesis [12, 13] has simultaneously increased cancer detection rate and in low-specificity programmes, it has reduced recall rates. None of the studies, to date, has been powered to measure interval cancer rates but initial reports have shown minimal if any reduction [14]. The information on grade distribution of the cancers detected suggests a preferential detection for lower grade disease [14, 15]. Both screen detection rates and interval cancer rates therefore indicate a high sensitivity for lower grade invasive cancers. However, if we are to further improve performance and increase mortality reduction, we need to understand the limitations of current technology and the ability of new technology to detect small grade 3 cancers.

A high sensitivity for a grade of cancer at screening is indicated by a high detection of small cancers of that grade and low detection of interval cancers of that grade. This paper examines 7 years of screening data from 2009/2010 to 2015/2016 to determine the invasive cancer detection rate by grade and size in the English NHS breast cancer screening programme (NHSBSP) with a particular emphasis on grade 3 invasive cancers. We use our large dataset to explore relative sensitivity using detection rates at prevalent (first) and incident (subsequent) screens. The former are particularly important as they are not influenced by screening interval and therefore can give greater insight into the detection of small grade 3 cancers using current technology.

## Methods

This study was undertaken using data from an ongoing population-based breast screening programme for asymptomatic women aged 50–70. Since 2010 as part of the AgeX trial [16], first invitations now occur in the age range 45–52 years. The NHSBSP uses a single national information technology system (NBSS) and collects standardised data on all breast screening activity which is published annually [17]. The NHSBSP has gained approval to access and process patient data for the purposes of quality control under section 251 of the Health and Social Care Act 2008 through the approval of the Confidentiality Advisory Group (previously the National Information Governance Board for Health and Social Care). Because this study did not involve patient contact, intervention or use of identifiable patient data, it was determined to be exempt from human subject’s ethical review in the UK. Ethics committee approval was therefore not required and the need for written informed consent was waived.

The NHSBSP is a large nationally organised programme where equipment and techniques are standardised. Women eligible for breast screening are identified from the NHS database and invited 3 yearly by one of the 80 breast screening facilities in England. Each facility has a static (hospital-based) unit and several mobile vans or satellite facilities each with a mammography unit. Bilateral two-view mammography is independently double-read by film readers with defined national standards for training, caseloads and performance. Arbitration is undertaken for cases where there is a discrepancy between the reader’s opinions. Women recalled for further investigation (assessment) attend the responsible screening service. Assessment is conducted according to the national guidance and all women undergoing biopsy are discussed at multidisciplinary team meetings [18, 19].

### Study population

Numbers of women screened, and cancers detected are taken from the National Korner (KC62) returns sent to Public Health England (PHE) annually for the 80 screening facilities followed over the seven screening years 2009/2010 to 2015/2016. Over the study period, it is estimated that 65% of mammograms were taken using digital mammography and the rest analogue screen film [11]. Information on the grade and size of invasive cancers is taken from the KC62 annex. The data used are for all routine prevalent (first) screens at ages 45–52 years and incident screens (subsequent) at ages 53–70 years to allow for greater comparability between facilities. All women who underwent their first screen after previously being invited and not attending were excluded. Interval cancer data for this period is not yet available so it is not possible to calculate absolute sensitivity, and estimates of relative sensitivity have been made using published data [8].

### Statistical methods

Statistical tests are given where considered useful. Funnel plots of individual unit data comparison show 95% and 99.8% control limits. All graphs and statistical analysis use STATA version 14.

## Results

Over the 7 screening years 2009/2010 to 2015/2016, there were a total of 11,258,620 eligible screening attendances and 86,443 (7.7 per 1000) cancers were detected of which 67,681 (6.0 per 1000) were invasive. Of the invasive cancers, 65,509 (97%) had both size and grade recorded.

### Detection rates by size and grade

**Fig. 1:**
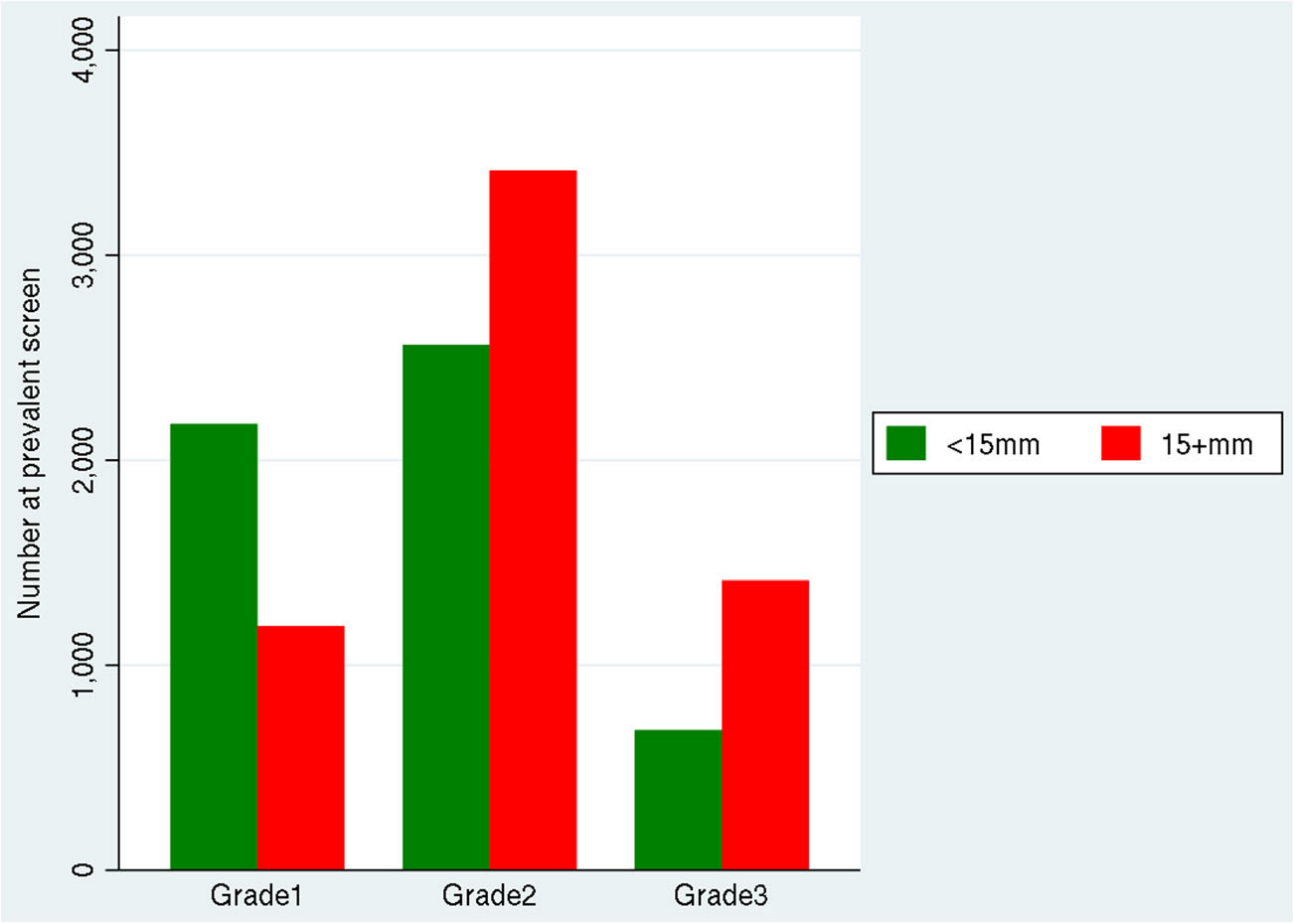
Number of prevalent screen invasive cancers by size (< 15 mm or 15 + mm) for each grade

Table 1 and Fig. 1 show details of the observed number and rate of invasive cancers with both size and grade is recorded. At prevalent screens, the rate of large (≥ 15 mm) invasive cancers was 0.52 per 1000 women for grade 1, 1.49 per 1000 for grade 2 and 0.62 per 1000 at grade 3 at age approximately 50 years, which is also the underlying rate of disease for large cancers assuming close to 100% sensitivity (see the “Discussion” section). The ratio of small to large invasive cancers is a key indicator of the ability of screening to detect small invasive cancers. At prevalent screens (which are not affected by screening interval), the detection rate of small grade 1 cancers (0.95 per 1000) is nearly double (1.8:1) that of large tumours (0.52 per 1000). However, this ratio reverses for grade 3 cancers (0.5:1) where large cancers at prevalent screens (0.62 per 1000) are found at over double the rate of small cancers (0.30 per 1000). This contrasts with the grade 1 data and suggests that there is a large pool of undetected small grade 3 invasive cancers and therefore that mammography has a much lower sensitivity for small grade 3 invasive cancers.

**Table 1:** Prevalent screen and incident screen association between size and grade for invasive cancers from 2,295,016 prevalent and 8,963,604 incident screens with number, percentage and rate per 1000 (based on 65,509 (97%) of 67,681 invasive cancers with both size and grade recorded)

	Grade 1		Grade 2		Grade 3		Total
	< 15 mm	15 + mm	< 15 mm	15 + mm	< 15 mm	15 + mm	
	*N* (%), rate p 1000	*N* (%), rate p 1000	*N* (%), rate p 1000	*N* (%), rate p 1000	*N* (%), rate p 1000	*N* (%), rate p 1000	*N* (%), rate p 1000
Prevalent	2177 (19.0), 0.95	1192 (10.4), 0.52	2562 (22.4), 1.12	3414 (29.8), 1.49	682 (6.0), 0.30	1414 (12.4), 0.62	11.441 (100), 4.99
Incident	10,245 (19.0), 1.14	3295 (6.1), 0.37	15,231 (28.2), 1.70	14,036 (26.0), 1.57	4467 (8.3), 0.50	6794 (12.6), 0.76	54,068 (100), 6.03
Total	12,422 (19.0), 1.10	4487 (6.8), 0.40	17,793 (27.2), 1.58	17,450 (26.6), 1.55	5149 (7.9), 0.46	8208 (12.5), 0.73	65,509 (100), 5.78

For incident screens, the detection of invasive cancers is a function of the numbers removed from the system by the earlier screens, the growth rate of the cancers and screening sensitivity. The highest detection rate at incident screens is for small (< 15 mm) grade 2 invasive cancers at 1.70 per 1000 and the lowest detection rate is for large (≥ 15 mm) grade 1 invasive cancers. At incident screens, the detection rate of small (< 15 mm) grade 1 invasive cancers is 1.14 per 1000 compared with 0.37 per 1000 for large grade 1 cancers (≥ 15 mm), a ratio of 3.1:1. The ratio is again reversed for grade 3 invasive cancers where it is 0.7:1.

**Fig. 2:**
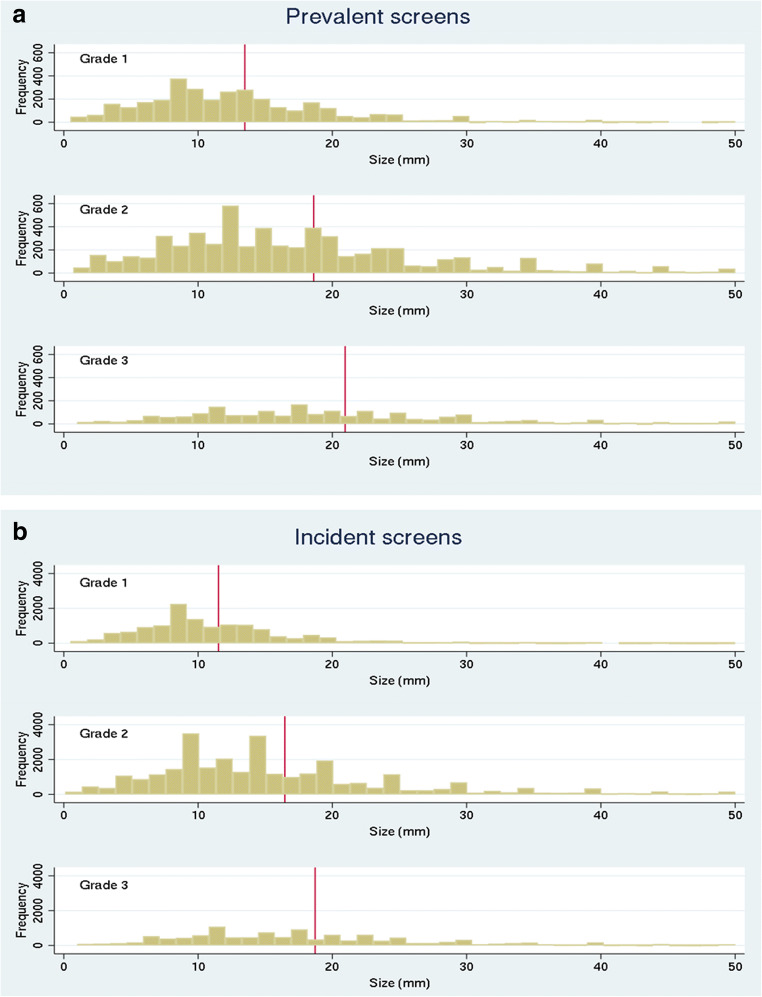
**a** Histogram of the size distribution of invasive cancers detected at prevalent screens by grade (mean size 13.7, 19.0 and 21.4, one-way ANOVA *p* < 0.001). **b** Histogram of the size distribution of invasive cancers detected at incident screens by grade (mean size 11.6, 16.7 and 19.0, one-way ANOVA *p* < 0.001)

To provide more detail, Fig. 2a and b show histograms of the size distribution of invasive cancers at prevalent and incident screens by grade with mean size at each grade shown by the vertical line. At prevalent screens, the mean size of a screen-detected grade 1 invasive cancer (13.7 mm) is less than the mean size of grade 2 invasive cancers (19.0 mm) and grade 3 invasive cancers (21.4 mm). These differences are highly significant (*p* < 0.001). Similar results are seen for incident screens where the mean sizes of grades 1, 2, and 3 invasive cancers are 11.6 mm, 16.7 mm and 19.0 mm respectively (*p* < 0.001). Very few grade 1 invasive cancers are detected at larger sizes, particularly at incident (subsequent) screens. Close inspection of the histogram shows that fewer grade 3 invasive cancers are detected at less than 10 mm for either prevalent (13%) or incident (16%) screens and this reduces to very low numbers at less than 5 or 6 mm. At prevalent screens, the percentage of invasive cancers of 1–5 mm is 12.1% for grade 1 cancers reducing to 7.4% for grade 2 and only 4.3% for grade 3. Prevalent screen detection rates should more closely reflect the distribution of underlying pre-clinical disease and the finding of so few very small grade 3 invasive cancers at prevalent screens suggests that grade 3 invasive cancers at 1–5 mm may therefore mostly be effectively occult.

#### Numerical estimates of relative disease sensitivity (from prevalent screen data)

The growth of breast cancers is usually modelled using exponential or Gompertz models [20]. Grade 3 invasive cancers grow faster on average than other invasive cancers, but the underlying model can be considered the same and therefore, the proportion below a certain size will be the same (see the “Discussion” section). To explore further, we make three assumptions about the distribution of these invasive cancers: (a) the relative size distribution of invasive cancers in unscreened women (i.e. the distribution in the pre-clinical detectable phase) should be approximately the same regardless of grade, (b) most invasive cancers of size 15 mm and above are detected by mammography and (c) that progression of cancers from lower to higher grades in these cancers is small. We can then use these assumptions to make a simple estimation of the relative sensitivity for grade 2 and grade 3 disease relative to grade 1 (Table 2) at prevalent screens. The ratio of < 15 mm to ≥ 15 mm grade 1 invasive cancers is 1.8:1 based on 2177 < 15 mm and 1192 ≥ 15 mm from Table 1). If we apply the same ratio to grade 2 and grade 3 invasive cancers, the estimated relative sensitivity for grade 2 relative to grade 1 is 62% and for grade 3 relative to grade 1 is 52%. Relative sensitivity for small (< 15 mm) grade 2 and 3 cancers is lower at 42% and 26% respectively. Table 3 shows our estimation of relative sensitivity along with estimations of relative sensitivity from published information adapted from Porter et al [8] and Perron et al [9] showing some agreement and therefore that such an approach could be useful.

**Table 2:** Prevalent screen association between size grade and nodal status and estimated sensitivity of grade 2 and 3 invasive cancers relative to the sensitivity for grade 1 invasive cancers

	Grade 1		Grade 2		Grade 3		Total
	< 15 mm	15 + mm	< 15 mm	15 + mm	< 15 mm	15 + mm	
Invasive cancers (row 1)	2177	1192	2562	3414	682	1414	11,441
Expected < 15 mm if the ratio is the same as grade 1 (row 2)	2177		6235 (= 3414 × 1.8)		2582 (= 2582–682)		
Undetected < 15 mm if ratio same as grade 1 (i.e. 1.8:1) (row 3)	0		3673 (= 6235–2562)		1900 (2582–682)		
Detective invasive cancers (row 4)	2177 + 1192 = 3369		2562 + 3414 = 5976		682 + 1414 = 2096		
Potential total if sensitivity is the same as for grade 1 (row 5)	3369		3693 + 5976 = 9649 (row 3 + row 4)		2096 + 1900 = 3996 (row 3 + row 4)		
Estimated sensitivity for invasive cancers relative to grade 1 (row 6)	100%		62% (5976/9649) 100% × row 4/row 5		52%(2096)/(3996) 100% × row 4/row 5		
Estimated sensitivity for invasive cancers (< 15 mm) relative to grade 1	100%		41% (2562/6235) 100% × row1/row2		26% (682/2582) 100% × row 1/row 2

**Table 3:** Screen-detected and interval cancers by grade reported by Porter [8] with our estimates of their absolute and relative sensitivity compared with our estimate of relative sensitivity from Table 2 and Perron [9]

	Porter et al 2007 (ref [8])					This study	Perron et al 2018 (ref [9])
Grade	Screen detected %	Interval%	Total	Estimated absolute sensitivity (%)	Relative sensitivity cf. grade 1 (%)	Relative sensitivity cf. grade 1 (%)	Relative sensitivity cf. luminal A
1	157 (34.6)	28 (9.7)	185	85	100	100	Luminal A 100%
2	172 (37.9)	120 (41.5)	292	59	69	62	Luminal B 88% Triple negative 70%
3	125 (27.5)	141 (48.8)	266	47	55	52	60% HER-2 positive
Total	454	289 (100)	743	61			

### Individual facility detection rates

**Fig. 3:**
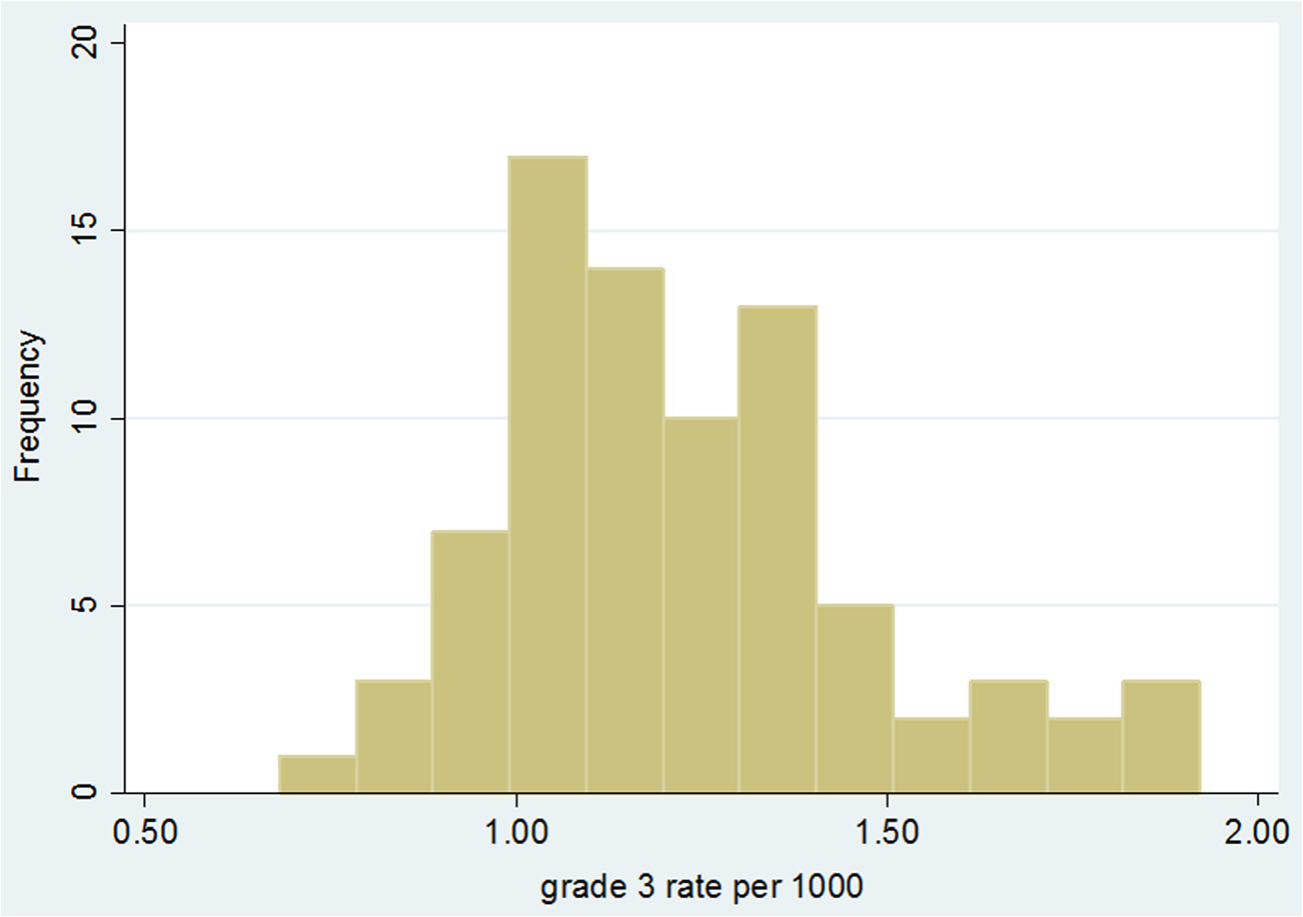
Histogram of grade 3 rate per 1000 for 80 screening facilities using data for prevalent and incident screens combined for the seven screening years from 2009/2010 to 2015/2016

Figure 3 shows a histogram of the individual facility grade 3 (prevalent and incident combined) invasive cancer detection rates for 80 screening facilities for the period 2009/2010 to 2015/2016. The mean rate was 1.22 per 1000 (SD 0.25) with a range of 0.68 to 1.92. The 25th and 75th percentiles were 1.05 and 1.34. In total, there were 13,693 grade 3 invasive cancers with 336 (2.5%) of unknown size. For cancers with known size, the mean detection rate of < 15-mm invasive cancers was 0.46 per 1000 (SD 0.12) with a range of 0.22 to 0.87 per 1000. The mean rate of ≥ 15-mm invasive cancers was 0.74 per 1000 (SD 0.18) with a range of 0.39 to 1.25 per 1000.

**Fig. 4:**
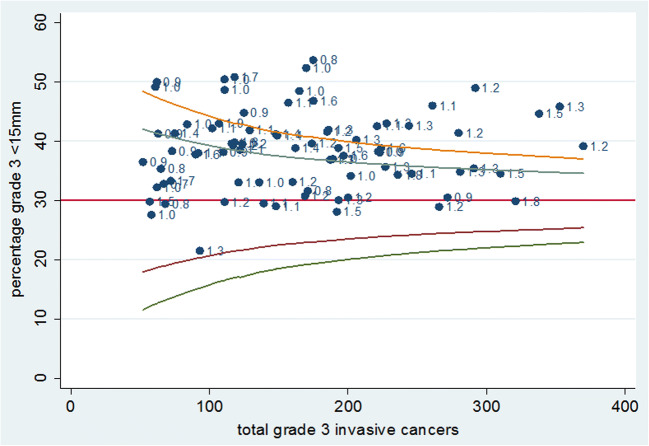
Funnel plot of percentage grade 3 cancers < 15 mm with 90 and 95% control limits around 30% and labelled with grade 3 detection rate for the 80 screening facilities for prevalent and incident screens combined for the seven screening years from 2009/2010 to 2015/2016

Figure 4 shows a funnel plot of the percentage of grade 3 invasive cancers with known size that are < 15 mm. This varies from 21.5 to 53.7% with a mean of 38.4%. Whilst some facilities have values below 30%, there are no facilities with a percentage of < 15-mm grade 3 invasive cancers below the lower 95% control limit of the 30% target suggested by Tabar et al [10]. There are also no facilities with exceptionally high values, the highest being around 50% and the distribution is normal around the mean of 38.4%. The facilities all converted to digital during the time period (mostly between 2010 and 2013) as per the policy of the national programme as film mammography units were replaced by digital mammography units gradually within each facility. There were some differences in the proportion of the study time period over which digital mammography was used by different units. However, as there was no difference in the detection of grade 3 cancers between film and digital [11], there would not be any confounding and the grade 3 detection rates between all units are therefore comparable.

## Discussion

### The importance of high-grade invasive cancer detection

Mammography screening is proven in multiple RCTs to reduce breast cancer mortality [21] and the UK Independent review estimates a 20% overall reduction in women invited [2]. The mortality reduction is closely related to the detection of grade 3 tumours and the Swedish two-county trial outweighed that from detecting grade 1 and 2 cancers combined despite fewer grade 3 cancers being detected [7]. High-grade tumours are over-represented within interval cancers [8, 22] and the rate of interval cancers in England remains at about 31 per 10,000 [23].

### Sensitivity for small high-grade cancers compared with historical data

In this retrospective observational study of the English Breast Screening Programme, we provide evidence of a relative lack of sensitivity for the detection of small grade 3 cancers estimated at only 26% of the sensitivity for small grade 1 tumours. The sensitivity of mammographic screening for these aggressive tumours may have changed little since the early RCTs of screening where the Swedish two-county RCT detected 37% of grade 3 cancers at less 15 mm [10]. Our data show a similar percentage (38.4%) and that whilst individual facilities tend to have greater than 30% of grade 3 invasive cancers detected at less than 15 mm, this target is based on mammography experience during the 1970s. There is no evidence provided by this data that the detection rate of grade 3 cancers is any better than that achieved in the 1970s. In contrast, we have strong evidence for substantial increases in the detection of high-grade DCIS, grade 1 and some grade 2 invasive cancers from technologies introduced since the beginning of programme such as two-view mammography and digital imaging [11, 24–26].

Relatively few grade 1 cancers are detected at incident screens which is likely to be a consequence of high detection of grade 1 tumours at prevalent screens and slow growth rate.

### Dataset

The origin of the data retrospectively from a national dataset does limit the breadth of data that is available. For instance, biomarkers and histological subtype categorisation such as triple negative are not available. Grade does however remain a strong prognostic indicator reflecting tumour aggressive potential [27] and the size of the dataset from a nationally organised programme where each site had similar techniques and equipment is powerful as large numbers of grade 3 cancers are required to produce the necessary level of statistical stability. In our dataset, 97% of invasive cancers had both grade and size recorded and the missing size data was evenly distributed between grade 1, 2 and 3 cancers suggesting the missing data is unlikely to skew results. Furthermore, with only 3% of invasive cancers not included in our main tables, rates will be only very marginally lower than if all invasive cancers had grade and size recorded.

#### Estimation of relative sensitivity at prevalent screen

Data on interval cancers for this period is not yet published so we have been unable to calculate absolute sensitivity by grade and size. We have estimated sensitivity relative to the detection of grade 1 cancers based on a number of assumptions. The first assumption is that the relative size distribution of tumours in an unscreened population is similar regardless of grade. We assume that the same model e.g. exponential model [20] applies to all grades independently and that the proportion below a particular size is always the same. The second assumption regarding detectability for all ≥ 15-mm cancers does not consider problems of masking by dense breast tissue [28, 29] and it is unlikely that exactly 100% of large cancers will be detectable. However, the cut-off size of ≥ 15 mm was chosen as it is a key performance indicator in the NHSBSP. This is because we have shown in earlier work that improvements in technology and reading protocols only resulted in the detection of invasive cancers < 15 mm [30]. The difference between higher and lower quality screening is therefore there in the detection of these small invasive cancers. Should a smaller (> 10 mm) or larger (>20 mm) cut-off be used, we have calculated that the change in the ratio of large to small grade 3 cancers would change by 5%. This would not change the overall premise of reduced sensitivity for grade 3 cancers. Our third assumption is that the rate of phenotypic drift from low to high grade in untreated primary cancers is small, unlike in treated metastatic or recurrent cancers [31].

A significant proportion of the conclusions drawn are made by extrapolating from the data; however, we believe that these assumptions are scientifically sensible. Furthermore, we have validated our estimate of a relative sensitivity of 62% (grade 2) and 52% (grade 3) using screen-detected and interval cancers from a large single English screening centre [8]. Crucially, this validation suggests that our assumptions are reasonable. Similar results relating to biomarkers are reported from a 2-year Canadian screening programme [9]. For prevalent screen data, we are suggesting that the rate of undetected (if we had the same sensitivity as for grade 1) small (< 15 mm) grade 3 invasive cancers is 1900 from 2,295,016 women which is 0.83 per 1000. There will also be 1.60 per 1000 undetected small (< 15 mm) grade 2 invasive cancers giving a total of 2.43 per 1000. We (RGB who calculated the original national interval cancers rate targets back in 1994) have estimated the expected interval cancer rate over the 3-year interval for prevalent screens only as around 2 per 1000. This is slightly lower than the 2.43 per 1000 and this may reflect the fact that some missed cancers will not occur as interval cancers, but be screen detected at the next screen. Finally, the purpose of this paper is twofold: firstly to present the data as in Table 1 and Fig. 1 and secondly to interpret that data. The data shown in Fig. 1 clearly show that small grade 3s are not being detected in large numbers and that this is independent of screening interval. This is an observation of fundamental importance to mammography screening.

### Changes in screening and cancer detection

The introduction of two-view mammography in the NHSBSP around the year 2000 increased the detection of invasive cancers by allowing indistinct features on one view to be resolved as irregular masses on a second ([26]. This resulted in a 15–20% reduction in interval cancers [25]. There has also been an increase in overall cancer detection by 14% with digital mammography, almost exclusively grade 1 and 2 invasive cancers, but no change in grade 3. There was a 39% increase in HNG DCIS detection. There is observational evidence suggesting that for every 3 extra cases of screen-detected DCIS, one interval cancer is prevented and that this association persists even after adjustment for small screen-detected cancers [11, 29, 32]. As our study is based on an estimated 65% digital usage [11] and the impact of digital is to increase detection of grade 1 and 2 but not grade 3 invasive cancers, then our estimates of the relative sensitivity of grade 3 invasive cancers at 100% digital usage could be considered marginally conservative.

### Challenges with detection of grade 3 cancers

The lack of overall change in grade 3 detection rates with the introduction of digital mammography and in the percentage of small grade 3 cancers over time implies that there has been little change in the last two decades in the detection of small high-grade invasive cancers. Grade 3 cancers accounted for 18.4% of the total invasive cancers detected in the prevalent round and 20.9% in the incident round. This contrasts with 48.8% of interval cancers being grade 3 [8], which is in line with a calculated relative screening sensitivity for high-grade cancers of around 50%. The radiological features of more aggressive cancers are described as subtle, including masses with indistinct margins and fine linear calcification. Round or oval aggressive malignant masses with well-circumscribed margins can mimic benign lesions [33, 34]. These features may explain the low sensitivity for high-grade tumours; i.e. fast-growing invasive cancers do not exhibit mammographic features such as calcifications associated with cancer [35]. In addition, the short doubling time of grade 3 lesions [36] may lead to them growing and becoming clinically detectable between screens and thus increase the likelihood of presentation as interval cancers. The majority of grade 3 invasive cancers therefore appear without any prior warning even if there is a prior screen. This tallies with the body of evidence that suggests that the majority of interval cancers are true interval or occult interval breast cancers that were not visible on the index screen [37]. We infer that many small grade 3 invasive cancers are mammography invisible; for example, they have the same density as the surrounding tissue and no additional features such as spiculations or calcifications.

### Generalisability of this study

The NHSBSP offers 3 yearly mammography which may increase both screen cancer detection rates and interval cancer rates in comparison with countries with annual and biennial screening programmes. The findings at the prevalent screen however will be generalisable to other mammographic screening programmes. Our findings at incident screens are not directly generalizable to screening programmes with different screen intervals. However, our findings may also be important because if small grade 3 invasive cancers are not detectable, then they will still be underrecognised using a 2-year or even a 1-year interval.

### The effect of recall rate

In England, observational data suggests increasing recall rate is not the answer to the poor sensitivity of mammography for high-grade cancers. There is a threshold maximum recall rate which is modelled to allow detection of 99% of the screen-detectable cancers in the NHSBSP, which is 7% at the prevalent screen and 4% at incident screen [38]. Above this recall rate, very few extra cancers are found, mainly low- and intermediate-grade DCIS.

#### Is there a role for new technology?

Despite the difficulties of finding small grade 3 cancers, mammography screening still reduces mortality. Any further improvement in mortality reduction requires finding proportionally more grade 3 cancers when small. This is doubly difficult because of morphology i.e. a lack of mammographic features when small and biology, as the doubling time of grade 3 cancers on mammography is approximately a third of the doubling time of grade 1 cancer with an average of 105 days vs average of 353 days [34] or 128 vs 194 [39].

To date, digital mammography has preferentially increased the detection of lower risk disease [11], but does bring the advantage of post processing which could be used to maximise soft tissue lesion detection [40]. Some types of post processing of digital raw images may favour the detection of spiculations and calcifications over soft tissue lesions and benefit the detection of lower, rather than higher grade invasive cancers, as well as ductal carcinoma in situ (DCIS). The selection of post processing algorithms to enhance soft tissue could lead theoretically to an increase in detection of subtle, but not necessarily high-risk malignancies.

Tomosynthesis certainly increases cancer detection, but the additional cancers are predominantly low grade [15, 41] and to date, there has been no impact on interval cancer rates [12, 42]. The additional detection of small grade 3 cancers is unclear, as grade size distribution has not been reported, but current evidence does not suggest that this is the answer to increasing the detection of small fast growing cancers destined to become interval cancers.

Supplemental ultrasound, which uses different biological imaging properties, for dense breasts identifies more cancers, but again these do not appear to be preferentially high grade resulting in minimal impact on interval cancer rates [43].

Functional imaging might help as interval cancers show higher mitotic rates and angiogenesis than screen-detected ones [22], and there is evidence that MRI is much better at detecting cancers of high nuclear grade [44]. Along with MRI, another promising new imaging method is contrast enhanced spectral mammography (CESM) which has a similar sensitivity and specificity to MRI [45].  The DENSE trial has not yet published grade size distribution but in those 10% of women with the highest breast density, there was a significant fall in interval cancer rates suggesting that MRI may be a more sensitive screening test for high-grade cancers. However, significant numbers of additional grade 1 and grade 2 together with benign and indeterminate lesions were also detected [46]. 

## Conclusion

This study has demonstrated poor relative sensitivity of screening mammography for the detection of small invasive grade 3 cancers. This is likely to be related to both the poor visibility of high-grade invasive cancers when small as well as the short doubling time. Despite the fact that this mammographic screening has shown a significant mortality reduction, any further improvement in technology or process needs to concentrate on the detection of smaller grade 3 cancers.

## Electronic supplementary material

ESM 1 (DOCX 32 kb)

## Abbreviations

DCIS: Ductal carcinoma in situ
HNG: High nuclear grade
NBSS: National Breast Screening System
NHSBSP: National Health Service Breast Screening Programme
PHE: Public Health England

## References

[1] Gøtzsche PC, Nielsen M (2011) Screening for breast cancer with mammography. Cochrane Database Syst Rev (1) The Cochrane Collaboration Wiley

[2] Marmot MG, Altman DG, Cameron JA, Thompson SG, Wilcox M (2013). The independent UK review panel on breast cancer screening. The benefits and Harms of breast screening: an independent review. Br J Cancer.

[3] Lauby-Secretan B, Scoccianti C, Loomis D et al (2015) Breast cancer screening The IARC working group. N Engl J Med 372:2353–2358 10.1056/NEJMsr1504363 26039523

[4] Welch HG, Prorok PC, O’Malley AJ, Kramer BS (2016). Breast-Cancer tumor size, overdiagnosis, and mammography screening effectiveness. N Engl J Med.

[5] Lannin DR, Wang S (2017). Are breast cancers good because they are small or small because they are good?. N Engl J Med.

[6] Tabár L, Dean PB, Chen TH (2019). The incidence of fatal breast cancer measures the increased effectiveness of therapy in women participating in mammography screening. Cancer..

[7] Tabar L, Chen THH, Yen AMF (2010). Effect of mammography screening on mortality by histological grade. Cancer Epidemiol Biomarkers Prev.

[8] Porter GJR, Evans AJ, Cornford EJ et al (2007) Influence of mammographic parenchymal pattern in screening detected and interval invasive breast cancers on pathologic features, mammographic features and patient survival. AJR Am J Roentgenol 188:676–683 10.2214/AJR.05.1950 17312053

[9] Perron L, Chang S-L, Daigle J-M (2019). Breast cancer subtype and screening sensitivity in the Quebec Mammography Screening Program. J Med Screen.

[10] Tabar L, Fagerburg G, Duffy SW, Day NE, Gad A, Grontoft O (1992). Update of the Swedish-two county programme of mammographic screening for breast cancer. Radiol Clin North Am.

[11] Blanks RG, Wallis MG, Alison R (2019). Impact of digital mammography on cancer detection and recall rates: 11.3 million screening episodes in the English National Health Service Breast Cancer Screening Program. Radiology.

[12] Li T, Marinovich ML, Houssami N (2018). Digital breast tomosynthesis (3D mammography) for breast cancer screening and for assessment of screen-recalled findings: review of the evidence. Expert Rev Anticancer Ther.

[13] Houssami N (2015). Digital breast tomosynthesis (3D-mammography) screening: data and implications for population screening. Expert Rev Med Devices.

[14] Lang K (2019). The coming of age of breast tomosynthesis in screening. Radiology.

[15] Skaane P, Bandos AI, Niklason LT (2019). Digital mammography versus digital mammography plustomosynthesis in breast cancer screening : the Oslo tomosynthesis trial. Radiology.

[16] http://www.agex.uk/ Accessed 1 Jan 2018

[17] NHS Digital, Breast Screening Programme, England - 2015-16 https://digital.nhs.uk/catalogue/PUB23376 Accessed 1 Jan 2018

[18] Service specification No. 24 NHS Breast screening Programme - NHS England London. https://www.england.nhs.uk/wp-content/uploads/2017/04/service-spec-24.pdf Accessed 1 Jan 2018

[19] (2011) Quality Assurance Guidelines for Breast Cancer Screening Radiology. NHS Cancer Screening Programmes, (NHSBSP Publication No 59)

[20] Peer GMP, van Dijck JAAM, Hendriks JHCL, Holland R, Verbeek ALM (1993). Age dependent growth rate of primary breast cancer. Cancer.

[21] Smith RA, Duffy SW, Gabe R, Tabar L, Yen AM, Chen TH (2004). The randomized trials of breast cancer screening: what have we learned?. Radiol Clin North Am.

[22] Gilliland FD, Joste N, Stauber PM et al (2000) Biologic characteristics of interval and screen-detected breast cancers. J Natl Cancer Inst 92(9):743–749 10.1093/jnci/92.9.743 10793111

[23] Burnside ES, Vulcan D, Blanks RG, Duffy SW (2018). The association between screening mammography recall rate and interval cancers in the UK breast cancer service screening programme: a cohort study. Radiology.

[24] Blanks RG, Bennet RL, Patnick J, Cush C, Davison C, Moss SM (2005). The effect of changing from one view to two views at incident (subsequent) screens in the NHS breast screening programme in England. Impact on cancer detection and recall rates. Clin Radiol.

[25] Dibden A, Offman J, Parmar D (2013). Reduction in interval cancer rates following the introduction of two view mammography in the UK breast screening programme. Br J Cancer.

[26] Given-Wilson RM, Blanks RG (1999). Incident screening cancers detected with a second mammographic view: pathological and radiological features. Clin Radiol.

[27] Arpino G, Milano M, De Placido S (2015). Features of aggressive breast cancer. Breast.

[28] Wanders JOP, Holland K, Veldhuis WB (2017). Volumetric breast density affects performance of digital screening mammography. Breast Cancer Res Treat.

[29] Destounis S, Johnston L, Highnam R, Arieno A, Morgan R, Chan A (2017). Using volumetric breast density to quantify the potential masking risk of mammographic density. AJR Am J Roentgenol.

[30] Blanks RG, Wallis MG, Moss SM (1998). A comparison of cancer detection rates achieved by breast cancer screening programmes by number of readers, for one and two view mammography: results from the UK National Health Service breast screening programme. J Med Screen.

[31] Hanby AM (2005). Aspects of molecular phenotype and its correlations with breast cancer behaviour and taxonomy. Br J Cancer.

[32] Duffy S, Dibden A, Michalopoulos D (2016). Screen detection of ductal carcinoma in situ and subsequent incidence of invasive interval breast cancer : a retrospective population-based study. Lancet Oncol.

[33] Woodard G, Ray K, Joe B, Price E (2018). Qualitative radiogenomics: association between oncotype DX test recurrence score and BI-RADS mammographic and breast MR Imaging features. Radiology.

[34] Gao B, Zhang H, Zhang S-D (2014). Mammographic and clinicopathological features of triple-negative breast cancer. Br J Radiol.

[35] Heuser LS, Spratt JS, Kuhns JG, Polk HC, Buchanan JB (1984). The association of pathologic and mammographic characteristics of primary human breast cancers with “slow” and “fast” growth rates and with axillary lymph node metastases. Cancer.

[36] Förnvik D, Lång K, Andersson I, Dustler M, Borgquist S, Timberg P (2016). Estimates of breast cancer growth rate from mammograms and its relation to tumour characteristics. Radiat Prot Dosimetry.

[37] Houssami N, Hunter K (2017) The epidemiology, radiology and biological characteristics of interval breast cancers in population mammography screening. NPJ Breast Cancer 3(12):1–13 10.1038/s41523-017-0014-x PMC5460204 28649652

[38] Blanks RG, Given-Wilson RM, Cohen SL, Patnick J, Alison RJ, Wallis MG (2019). An analysis of 11.3 million screening tests examining the association between recall and cancer detection rates in the English NHS breast cancer screening programme. Eur Radiol.

[39] MacInnes EG, Duffy SW, Simpson JA (2020). Radiological audit of interval breast cancers: estimation of tumour growth rates. Breast.

[40] Warren LM, Halling-Brown MD, Looney PT (2017). Image processing can cause malignant soft-tissue lesions to be missed in digital mammography images. Clin Radiol.

[41] Johnson K, Zackrisson S, Rosso A (2019). Tumor Characteristics and Molecular Subtypes in Breast Cancer Screening with Digital Breast Tomosynthesis: The Malmö Breast Tomosynthesis Screening Trial. Radiology.

[42] Hovda T, Holen AS, Lang K et al (2019) Interval and consecutive round breast cancer after digital breast tomosynthesis and synthetic 2D mammography versus standard 2D digital mammography in BreastScreen Norway. Radiology 294(2). 10.1148/radiol.2019191337 10.1148/radiol.2019191337 31821118

[43] Tice JA, Kerlikowske K (2017). Supplemental breast cancer screening: a density conundrum. J Gen Intern Med.

[44] Kuhl C, Strobel K, Bieling H, Leutner C, Schild H, Schrading S (2017). Supplemental breast MR imaging screening of women with average risk of breast cancer. Radiology.

[45] Patel BK, Lobbes MBI, Lewin J (2018). Contrast enhanced spectral mammography: a review. Semin Ultrasound CT MRI.

[46] Bakker MF, de Lange SV, Pijnappel RM (2019). DENSE trial study group. Supplemental MRI screening for women with extremely dense breast tissue. N Engl J Med.

